# Neurotoxicity of Inhalation Anesthetics in the Neonatal Rat Brain: Effects on Behavior and Neurodegeneration in the Piriform Cortex

**DOI:** 10.1155/2018/6376090

**Published:** 2018-06-19

**Authors:** Rachel A. O'Farrell, Andrew G. Foley, Donal J. Buggy, Helen C. Gallagher

**Affiliations:** ^1^Department of Anaesthesia, Mater Misericordiae University Hospital, Eccles Street, Dublin 7, Ireland; ^2^School of Medicine, Conway Institute, University College Dublin, Belfield, Dublin 4, Ireland; ^3^Bon Secours Hospital, Glasnevin, Dublin 9, Ireland; ^4^Berand Neuropharmacology, NovaUCD, University College Dublin, Belfield Innovation Park, Dublin 4, Ireland; ^5^Outcomes Research Consortium, Cleveland Clinic, Cleveland, OH, USA; ^6^UCD-Mater Clinical Research Centre, Eccles Street, Dublin 7, Ireland

## Abstract

There is concern that clinical use of anesthetic drugs may cause neurotoxicity in the developing brain and subsequent abnormal neurobehavior. We therefore evaluated neurotoxic effects of inhalation anesthetics in the neonatal rat brain, using in vivo histological and neurobehavioral outcomes. Wistar rats (*n*=79, postnatal day 15) were subjected to a clinically relevant single exposure of urethane, isoflurane, sevoflurane, or placebo, without surgery. At 48 h and 96 h, behavioral parameters were recorded and the animals were sacrificed. In cryosectioned brains, total cells and dying cells in layer II of the piriform cortex were counted using unbiased stereology. At 48 h, cell numbers in layer II of the piriform cortex of all drug-treated animals were reduced versus controls (*p*=0.01). The effect persisted at 96 h in isoflurane- and urethane-exposed animals. Piriform cortical layer II neurons undergoing degeneration, detected histologically by pyknotic nuclei and eosinophilic cytoplasm, were increased in the animals treated with isoflurane (1.9 ± 0.7 at 96 h) and urethane (2.4 ± 0.8 at 96 h) versus sevoflurane (0.8 ± 0.3 at 96 h) and controls (0.9 ± 0.2 at 96 h). Sevoflurane- and isoflurane-treated animals exhibited increased activity and decreased suckling compared with controls, and sevoflurane-exposed animals also displayed increased rearing behavior at both timepoints.

## 1. Introduction

There is accumulating evidence that, during synaptogenesis, the brain is sensitive to toxicity from many environmental agents, potentially resulting in neurological injury manifesting as neuroapoptosis [1]. Of those prescribed drugs in common clinical use, concerns particularly abound in relation to anesthetic, sedative, and analgesic agents. Drugs from these classes implicated in causing neurodevelopmental toxicity include ketamine, volatile anesthetics, nitrous oxide, propofol, and barbiturates, all of which are commonly employed in emergency medicine and/or in routine surgery [2–10]. In an early paper, the prototypical anesthetic urethane, no longer in clinical use, caused significant apoptosis in the piriform cortex, when given as a single dose to two-week-old animals [11]. The piriform cortex is a crucial structure for neuromodulatory effects on cortical processing, with an established role in both memory processing and behavior [12]. Since both perioperative cognitive dysfunction and behavioral anomalies, such as emergence agitation, are of concern in clinical anesthesia, this cortical region is of particular interest from a neurotoxicological perspective [13–15]. In clinical practice, urethane and halothane have been superseded by the newer, and supposedly safer, fluorinated inhalational agents: sevoflurane, isoflurane, and desflurane. However, the effects of these drugs on the piriform cortex have not been reported.

The importance of evaluating experimental and preclinical data on the extent and significance of anesthetic-induced neurotoxicity has recently propelled this issue to the forefront of research in anesthesiology prompting the US Food and Drug Administration (FDA) to establish an Expert Working Group [16–18]. We previously demonstrated that isoflurane and enflurane inhibit neural cell proliferation in vitro, whereas sevoflurane was much less antiproliferative [19]. The neuroapoptotic properties of modern inhaled anesthetics have been described in other brain areas [20, 21], and in mice, recent studies have reported long-term consequences of isoflurane on spatial memory [22] and of desflurane on working memory [23]. However, there is a paucity of information correlating the short-term behavioral and postmortem histological neuroapoptotic sequelae of these agents. Moreover, the topography of anesthetic-induced neurotoxicity is not well defined. Here, therefore, we compared the effects of a single, clinically relevant exposure to urethrane, isoflurane, or sevoflurane in postnatal day 15 rats, in terms of behavior and piriform cortical neuronal histology postexposure.

## 2. Methods

### 2.1. Animals

We deliberately chose to use postnatal day 15 (PD15) male Wistar rats for all experiments in an effort to explore the link between piriform cortical neurotoxicity and neurobehavioral outcomes in an animal model exposed to clinically relevant concentrations of anesthetic agents. While PD15 immediately follows the most prominent brain growth spurt in rats, it is very difficult to perform behavioral studies in younger animals. All animals were administered either an experimental drug or placebo. They were then allowed to recover in the home cage for either 48 hours or 96 hours. Behavioral data were collected, and the animals were sacrificed. Their brains were extracted and stored for later analysis. The experimental procedures were approved by the Animal Ethics Review Committee of the Biomedical Facility, University College Dublin, where these animals were bred, and were carried out by an individual who held the appropriate license issued by the Irish Minister of Health and Children.

### 2.2. Anesthetic Exposure

The animals were administered a single dose of anesthetic via a route appropriate to the agent employed (subcutaneous injection or inhalation), with vehicle-treated animals serving as controls. Urethane, isoflurane, and sevoflurane were tested for potential neurotoxicity, and the animals were exposed to these agents at drug concentrations and doses approximated to the clinical setting. The primary reason for using urethane as a positive control is that it is a prototypic agent that is well known to be neurotoxic to the piriform cortex [11]. Specifically, each animal in the urethane study group (*n*=21) received 1.25 g/kg urethane as a single subcutaneous dose and was then placed in the anesthetic delivery box and administered air/oxygen at a fractional inspired oxygen (FiO_2_) concentration of 0.5, until recovery from anesthesia. Urethrane at this dose produces general anesthesia in these animals, with 30 min being the longest recovery time. The isoflurane and sevoflurane animals were placed in the anesthetic box and administered an air/oxygen/vapor admixture for 4 hours and then allowed to recover with an FiO_2_ of 1.0. They were spontaneously breathing throughout.

Depth of anesthesia was determined by an a priori calculation of the inhaled volatile agent MAC, with measurement of end-tidal concentration to ensure that this MAC was being delivered. In the rat, minimum alveolar concentration (MAC) can be determined using the tail-clamp technique. There is an age-dependent relationship with respect to anesthetic requirements, with MAC being significantly higher in younger animals. The most up-to-date values for MAC in the neonatal rat are from Orliaguet and colleagues [24]. Based on this work, we determined that the most appropriate isoflurane MAC value for a PD15 rat would be 2.08% and for sevoflurane 3.46%. Thus, the end-tidal isoflurane (Forane, Abbott Laboratories, Ireland) concentration was maintained at 2.1% (*n*=18) and end-tidal sevoflurane (Sevorane, Abbott Laboratories, Ireland) at 3.4% (*n*=18), with an FiO_2_ of 0.5. All animals had an FiO_2_ = 0.5. An infrared gas analyzer (Datex Ultima, Helsinki, Finland) was used in all experiments, with the gas sampling line taped in a position adjacent to the animals' snouts. To maintain normothermia, all anesthetized animals were placed on a prewarmed electric heating blanket (Harvard Apparatus homeothermic blanket) and their body temperature maintained at 37°C. Control animals for the urethane group (*n*=10) were given a saline injection and placed in the box, and control animals for the sevoflurane and isoflurane groups (*n*=12) were given an air/oxygen mixture for 4 hours.

### 2.3. Behavioral Analysis of Neurotoxicity

To determine behavioral manifestations of any possible acute developmental insults to the brain, behavioral parameters were collected for each animal prior to sacrifice, that is, on day 2 or day 4 after anesthetic exposure. The tests used in this experiment were based on available recommendations for the behavioral assessment of functional neurotoxicity in young rats [25]. The observer was blinded to the group allocation of the animal being assessed. Of note, due to the young age of the rat pups, motor activity was assessed in activity boxes rather than in an “open-field”-type environment [26]. The animals were studied for just 5 minutes, as preweaning pups cannot be separated from the dam for longer periods of time to avoid maternal separation distress. Behavioral endpoints assessed were locomotion/activity (the number of independent movements and the percentage of time active), grooming (the amount of time the animal spent grooming), rearing, pivoting, and vocalization. For each parameter, the number of times each of these behaviors occurred in the observation period was counted. After each animal from a specific litter had been assessed individually in the activity box, all of the pups were returned to the home cage simultaneously. Home cage activity was then observed and recorded for a further 30 minutes. Specific endpoints assessed were based on previously published evaluations of maternal-pup interaction, including retrieval efficiency, maternal grooming, vocalization, suckling score, and maternal resting time.

### 2.4. Histopathological Studies

The ultimate goal of this study was to quantify the total number of cells and the number of dying cells in a specific layer of the piriform cortex of animals who had received a study drug and to compare these counts with those of the control animals. Thus, the total number of cells in specific preassigned areas of sections of the piriform cortex was counted in 6 alternate 30 *µ*m sections commencing −8.1 mm from Bregma. In this experiment, hematoxylin and eosin (H&E) staining of the brain tissue was employed to perform cell quantification. Using Brown and Brierley's description of irreversible cell injury [27], the brains were examined for evidence of dying cells after anesthetic exposure. The total number of dying cells was compared between the experimental animals and the controls and between the different drug groups. The use of eosinophilia as a criterion for irreversible brain damage at the light microscope level has been validated and avoids the pitfall of “dark cell” artifacts [11, 28].

Each animal was sublethally anesthetized with sodium pentobarbitone (Euthatal, Merial Animal Health Ltd., Dublin; 200 mg/ml) at a dose of 50 mg/Kg. A deep surgical plane of anesthesia was identified when there were no response to deep stimulation, absent corneal and righting reflexes, and a shallow breathing pattern. The heart was then cannulated, and the brain was perfused and fixed with 4% buffered paraformaldehyde. After extraction of an animal brain from its skull, it was immediately placed in a specimen tube containing 30% sucrose, a cryoprotectant, and stored for 48 hours at 4°C. They were then “snap-frozen” in precooled *n*-hexane after being coated with optimal cutting temperature (OCT) embedding medium (Lab-Tek Products, Miles Laboratories, IL, USA) and stored at −80°C until sectioning. To section the fixed cryoprotected brains, a sliding cryomicrotome was used, that is, a “cryostat” (Microm Series 500, Microm International, Germany). The brain was sectioned down to the level of the piriform cortex, and 12 alternate 30-micron sections were taken through it. For H&E staining, we chose the “regressive” method, which achieves a greater depth of staining [29]. This required the stronger form of hematoxylin, that is, “Harris hematoxylin” (Sigma Diagnostics, Missouri, USA).

For differentiation, we used acid alcohol 1%. “Eosin Y aqueous” (Sigma Diagnostics, Missouri, USA) was then applied. Eosin Y is used as a fluorescent indicator, and its fluorescence can be seen with dark-ground illumination, without special filters. Stained sections were mounted with “Citifluor” (Agar Scientific, UK).

For cell counts, three anatomical regions of layer II of the piriform cortex on each side of the brain were selected, as this location has previously been described as being a particularly vulnerable area of the developing brain for anesthetic-induced neurotoxic injury [11]. All sections were first evaluated under regular light to measure the total cell count and then under ultraviolet (UV) light with a fluorescein filter to evaluate fluorescence and identify cells undergoing degeneration. Quantitative image analysis was performed using the Leica Quantimet 500^®^, a PC-based software package, which was connected to the fluorescence microscope with a high sensitivity charge-coupled device (CCD) video camera (AxioCam HR, Zeiss, Germany).

The grid section that was used for counting cells was 60,000 *µ*m^2^, and since the sections were 30 *µ*m thick, each segment counted had a volume of 180,000 *µ*m^3^. This 60,000 *µ*m^2^ grid was placed over each of the six preassigned areas (i.e., I–VI) of layer II of the piriform cortex, and the total number of granule cells seen under regular light in each of these field areas was counted, that is, six separate grid areas were counted along the piriform cortex, each of 60,000 *µ*m^2^ area. For each separate plane examined (i.e., I–VI), the mean number of cells was calculated using the 6 individual cell counts from the six separate sections. Thus, there was a mean value for region I, region II, region III, and so on. These 6 mean values were then used to generate an overall mean value for cell count for each specific brain. Thus, a mean value ± SEM for the total cell count for layer II was calculated, and this was then used to generate a mean cell density for each brain, by dividing this mean cell count by the volume of the cortex measured for each of the values used to calculate the mean, that is, 180,000 *µ*m^3^.

Since six segments of piriform were counted in each brain section, the total volume of the piriform cortex analyzed per brain section was 10,800,000 *µ*m^3^. The mean value obtained for the cell density for each brain was then multiplied by the total granule cell layer volume of the piriform cortex to obtain the absolute cell number per piriform cortex by applying Cavalieri's method [30], that is, the cell density multiplied by the granule cell layer volume will generate the total cell number per piriform cortex for each brain. The mean values from each of the experimental brains were then used to generate the mean ± SEM and the number of cells per layer II of the piriform cortex for each animal study group.

The frequency of injured cells in layer II of the right and left piriform cortexes was then assessed. The total number of dying cells in each of the 6 segments as described above, in each of the six sections, from each of the brains was counted. These damaged granule cells were initially identified under regular light, with their pyknotic nuclei and eosinophilic cytoplasm, and then confirmed under UV light by their fluorescent yellow cytoplasm. The total number of dying cells per piriform cortex was then calculated for each brain and thus for each study group using the Cavalieri principle.

Hence, using measured cell counts, mean ± SEM values were calculated for each brain with the results expressed as the (a) total cell count per piriform cortex and (b) total eosinophilic cells per piriform cortex. These results were then used to generate the mean ± SEM values for each animal group. For statistical analysis, 6 brains were counted from each of the 10 study groups.

### 2.5. Data Analysis and Statistics

Data were entered in a database using GraphPad Prism v.4.0 (Graph Pad Inc., San Diego, CA) and examined for distribution using the Kolmogorov–Smirnov test. Normally distributed parametric data were compared using analysis of variance with post hoc Dunnett's test for a defined control group. The Wilcoxon rank test was used for comparison of all the groups when data were not normally distributed. Data were expressed as mean ± SEM, and *P* < 0.05 was deemed statistically significant.

## 3. Results

### 3.1. Animal Behavior

**Figure 1:**
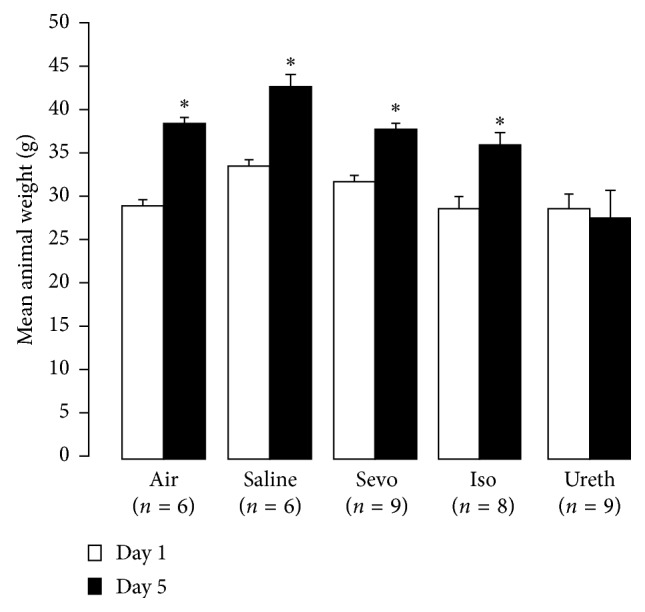
Animal weights on day 1 and day 5 after anesthetic exposure. The indicated treatments were administered in a single dose to PD15 rats for 4 hours (for inhalational agents) or as a single IV bolus (urethane), as indicated in Methods. Data represent mean ± SEM, with the number of animals indicated on the graph. Control animals were treated with air or saline, depending on the delivery route. Symbols indicate day 5 values that differ significantly from the day 1 values for that group (*P* < 0.05). Only the urethane-treated animals failed to gain weight over the analysis period. Iso: isoflurane; Sevo: sevoflurane; Ureth: urethane.

Data were collected on a total of 69 animals, out of a possible 79 experimental animals (this was due to 6 animals not surviving anesthesia and 4 animals being omitted from the behavioral stage in error). Of the six animals that died under anesthesia, 2 were from 18 animals under isoflurane anesthesia and 4 from 21 under urethane anesthesia. No animals died under sevoflurane anesthesia. The animals were weighed at the start of the study on day 1 and on day 5 prior to sacrifice (i.e., the 96-hour group). As expected, the control animals gained weight over the experiment duration, as did the sevoflurane-exposed and isoflurane-exposed animals. The urethane animals however failed to thrive and had no significant weight gain over the 5-day experiment duration (28.81 ± 5.47 g versus 27.5 ± 8.87 g, *P*=0.25; Figure 1).

To determine behavioral manifestations of any possible acute developmental insults to the brain, behavioral parameters were collected for each animal prior to sacrifice, as described in Methods. An individual behavioral data sheet was filled out for each animal, and the animal's behavior was assessed immediately prior to terminal anesthesia, that is, day 2 or day 4 after anesthetic exposure. This two-stage behavioral assessment comprised 5-minute observation in a Perspex activity box and 30-minute observation of maternal-pup interaction in the home cage. Parameters assessed were as follows:

  Locomotion/activity
  Grooming
  Rearing
  Pivoting
  Vocalization
  Retrieval efficiency
  Maternal grooming
  Suckling score
  Maternal resting time

Overall, the behaviors observed in control and treated groups fell within normal limits. However, some parameters displayed a statistically significant change in drug-treated animals, when compared to control animals (Table 1). Affected parameters included the following:

  Locomotor activity at 48 and 96 hours
  Grooming at 48 hours
  Rearing at 48 and 96 hours
  Suckling at 48 and 96 hours

Specifically, in sevoflurane-exposed animals, locomotion was not different at 48 hours when compared to controls; however, at 96 hours, there was a statistically significant increase in animal activity (Table 1). Rearing significantly increased at both 48 and 96 hours (Table 1). The suckling score significantly decreased in sevoflurane-treated animals at both 48 and 96 hours (Table 1). Isoflurane-exposed animals displayed an activity score similar to sevoflurane-treated animals at 48 and 96 h, but unlike sevoflurane-treated animals, those treated with isoflurane had comparable rearing scores to controls at both timepoints. Suckling scores significantly decreased at 96 hours only (Table 1).

In urethane-exposed animals, activity significantly decreased at both 48 and 96 h, as was rearing at 48 h when compared to control animals. However, rearing was comparable to controls at 96 h. Suckling scores were similar to controls at both timepoints; however, suckling time significantly increased in the urethane-exposed animals (*P*=0.03) compared to all other study animals.

### 3.2. Piriform Cortical Cell Counts and Apoptosis

**Figure 2:**
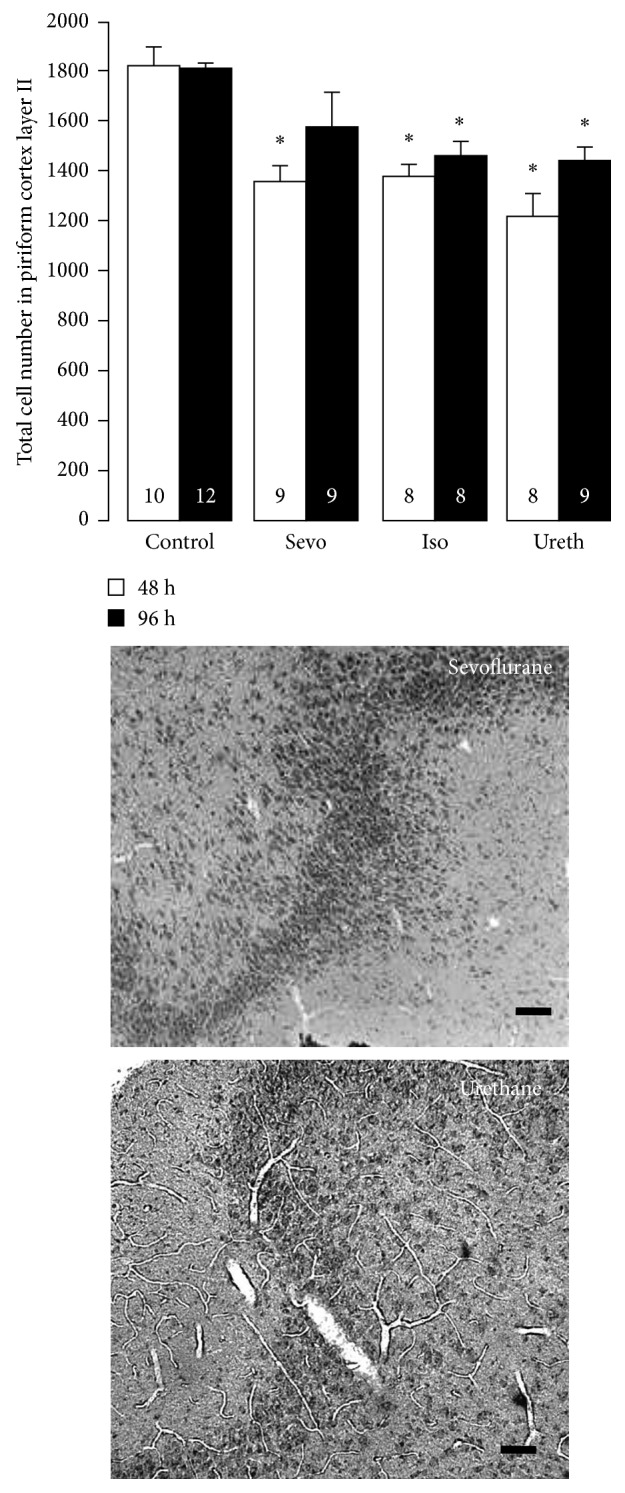
Neuronal integrity in the piriform cortex following anesthetic exposure. Data represent the mean ± SEM of total cell numbers in layer II of the piriform cortex in animals sacrificed 48 h and 96 h after anesthetic exposure on PD15. Values were calculated using unbiased stereological techniques, and those differing significantly from vehicle-exposed controls are indicated by an asterisk (^*∗*^*P* < 0.05). Representative images of H&E stained sections of the piriform cortex (layer II) are also shown (scale bar = 100 *µ*M). Pictures show the characteristically dense layer II, which is occupied primarily by the somata of pyramidal cells that receive direct input from the olfactory bulb upon their apical dendrites in layer I. These images are from animals sacrificed 96 h following anesthetic exposure. Iso: isoflurane; Sevo: sevoflurane; Ureth: urethane.

Total cell counts were calculated for layer II of the piriform cortex of each of the drug-treated animals, and a mean ± SEM value was calculated for each study group. When these were compared to those of the control brains, a statistically significant reduction was seen in all three drug groups (*P*=0.03 for sevoflurane and isoflurane and *P*=0.02 for urethane) at 48 h (Figure 2). However, in animals that had recovered for 96 h, there was no significant difference seen in the total cell count for the sevoflurane-exposed animals compared to controls. There was a significant reduction however in the number of total cells in the brains of those animals treated with isoflurane and urethane at 96 h (*P*=0.03). Of note, there was no statistical difference in cell counts between urethane and isoflurane at 96 h (Figure 2).

**Figure 3:**
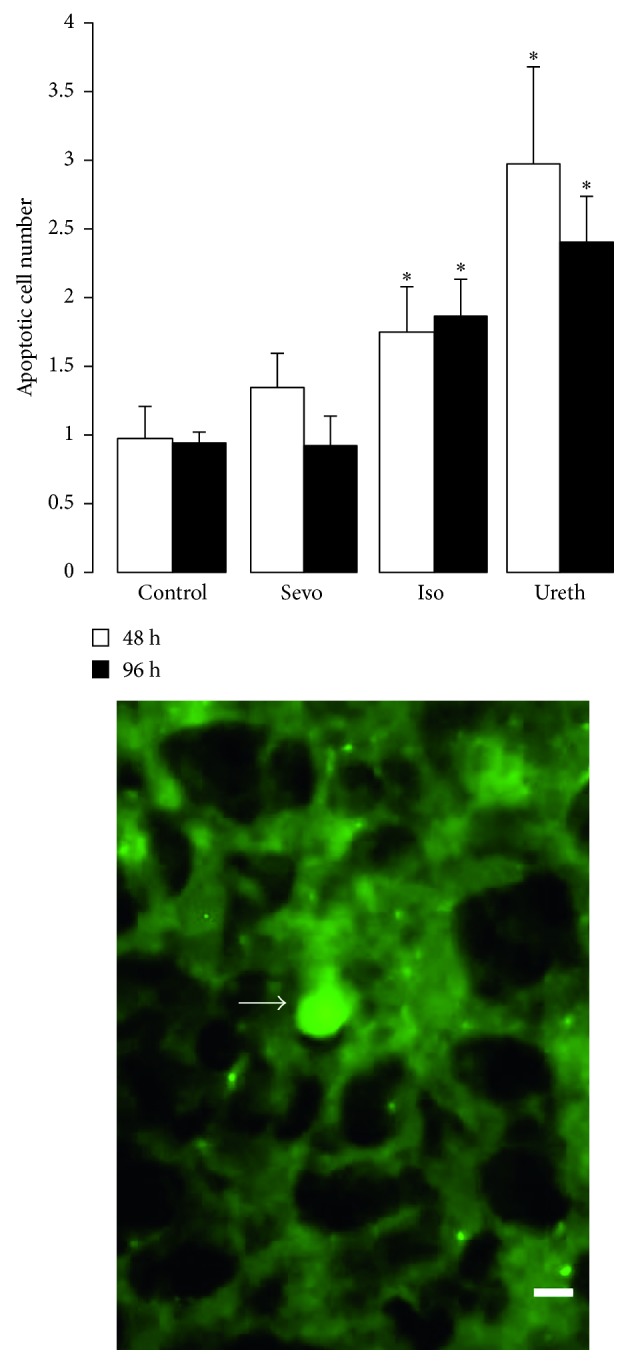
Neurodegeneration in the piriform cortex following anesthetic exposure. Data represent the mean ± SEM of total dying cells in layer II of the piriform cortex in animals sacrificed 48 h and 96 h after anesthetic exposure on PD15 (numbers of animals as per Figure 2). Dying cells were initially identified in sections stained with H&E, as having a shrunken pyknotic nucleus set in an intensely eosinophilic cytoplasm. In serial eosin Y-stained sections, they also fluoresced brightly under UV light. The number of dying cells was calculated using unbiased stereological techniques, and those differing significantly from vehicle-exposed controls are indicated by an asterisk (^*∗*^*P* < 0.05). Representative eosin Y-stained section of the piriform cortex (layer II) is shown under UV microscopy with a fluorescing, dying cell indicated by an arrow (scale bar = 20 *µ*M). The image is from an animal sacrificed 96 h following isoflurane exposure. Iso: isoflurane; Sevo: sevoflurane; Ureth: urethane.

Mean values were generated for each of the study groups for the number of dying cells per piriform cortex, and drug groups were compared to controls. In comparison with controls, there was a statistically significant increase in the number of apoptotic cells in the isoflurane- and urethane-exposed brains at both timepoints, that is, at 48 and 96 h (*P*=0.04 for isoflurane and *P*=0.02 for urethane; Figure 3). This effect was significantly more pronounced for urethane at both timepoints. Notably, the numbers of apoptotic cells in the brains of the sevoflurane animals were comparable to those of placebo controls (Figure 3).

## 4. Discussion

We have demonstrated in this in vivo rat model of the neonatal developing brain that a single 4-hour exposure to isoflurane produces piriform cortical toxicity similar to that reported previously with the antiquated anesthetic, urethane. Sevoflurane, in contrast, was shown to induce significantly less neurotoxicity, which recovered to baseline at 96 hours. Thus, although our data showed that all three anesthetic drugs cause some degree of neurological injury, sevoflurane appears to be least neurotoxic. This is consistent with our previous in vitro observation that isoflurane and enflurane produce pronounced antiproliferative effects in comparison with sevoflurane [19]. It is further supported by studies in the neonatal mouse, indicating that sevoflurane alone does not produce a robust neuroapoptosis when compared to effects seen when it is combined with propofol [23] and by the observation that a 4-hour exposure to isoflurane induced neuronal cell death in neonatal (postnatal day 7), but not aged, rats [31, 32]. Moreover, in a recent study, which directly compared neonatal exposure to sevoflurane and isoflurane in terms of long-term effects on memory, it was suggested that a single 4-hour exposure to isoflurane was more detrimental than sevoflurane in very young animals [33]. Another recent study showed that rats acutely exposed to sevoflurane for just 30 minutes at PD7 or PD15 exhibited short-term changes in dendritic spine densities but no lasting effects on memory or overall motor function, assessed three months later [34].

The piriform cortex receives input from the olfactory bulb and sends efferents to the hippocampus via the entorhinal cortex. It thereby connects the two canonical neurogenic regions of the adult rodent brain. For over two decades, this cortical region has been known to contain a population of neurons immunoreactive for markers of neuroplasticity including the polysialylated neural cell adhesion molecule, which is usually highly expressed in newly generated neurons [35]. More recently, these cells have been described as a population of immature neurons, arising during embryonic development, which lack synapses and remain in an immature state during postnatal development and into adulthood [36, 37]. It is thought that they constitute a reservoir of “plastic” neurons for recruitment into preexisting neural circuits. This could explain how the piriform cortex appears to be exquisitely sensitive to neurotoxic insult, such as that mediated by anesthetic drugs, but also capable of some recovery as we saw with sevoflurane.

The reduced neurotoxicity associated with the newer agent, sevoflurane, may reflect its lower solubility in blood [38], which is associated with rapid uptake and recovery compared to isoflurane. Rapid clearance may further explain its more favorable neurotoxicological profile, with neuronal cell loss evident at 96 h following the exposure to sevoflurane. Moreover, we did not observe any increase in numbers of dying neurons at either 48 or 96 h in the sevoflurane-treated animals. Thus, cell loss may represent transient suppression of cell proliferation or neuroplasticity in this region, as opposed to cell death. This idea is supported by our earlier in vitro work, which found a decrease in cell numbers after exposure to sevoflurane but no change in lactate dehydrogenase (LDH) activity—an indicator of cytotoxicity [19]. Notably, in vitro studies evaluating the mechanism of cell death induced by inhalational anesthetics have not proven to be conclusive [39–41].

In contrast, the isoflurane- and urethane-treated animals did have evidence (albeit limited) of neuroapoptosis. However, it is obvious that the small number of dying cells we counted four days after anesthetic exposure could not, in itself, account for the large amount of cell loss we documented. Interestingly, Eidt et al. demonstrated that cell death in the rat piriform cortex peaks during 12–24 h following a neurological insult (status epilepticus) and that, after this period, very few dying cells are apparent at any given timepoint, despite a significant loss of neuronal integrity [42]. It is, therefore, possible that we have missed the main wave of neurodegeneration by electing to examine animals at 48 h and 96 h following anesthetic exposure.

It is generally accepted that all general anesthetics in current clinical practice have either *N*-methyl-D-aspartate (NMDA) receptor antagonist or *γ*-aminobutyric acid_A_ (GABA_A_) receptor modulating properties. With respect to current theories concerning how such anesthetic drugs can produce neuroapoptosis during synaptogenesis, it is known to involve the translocation of Bax protein to mitochondrial membranes, where it disrupts membrane permeability, allowing extramitochondrial leakage of cytochrome c, followed by a sequence of changes culminating in activation of caspase-3 [43]. Upstream pathways, through which the signal is relayed from the cell surface GABA_A_ and NMDA receptors to Bax protein, have also been elucidated in [44].

In chicken B lymphocytes, isoflurane triggers apoptosis by activating the endoplasmic reticulum (ER) membrane inositol 1,4,5-triphosphate (IP_3_) receptors, which results in excessive calcium release from the ER [45]. In the same study, caspase-3 levels were measured after isoflurane exposure to confirm that the mechanism of cell damage was indeed apoptosis. When sevoflurane and desflurane were added, all 3 agents induced cell damage, as determined by the markers annexin V and propidium iodide, but sevoflurane and desflurane caused significantly less damage than isoflurane and were much less potent [45]. Only isoflurane resulted in activation of caspase-3. Sevoflurane and desflurane did not cause activation of caspase-3, a marker of apoptosis, which would appear to support our observations.

Our behavioral data demonstrated decreased activity in the urethane animals at 48 hours and 96 hours. These effects, along with the observed decrease in rearing and grooming, are typical manifestations of drugs that induce gross toxicity and most likely correlate with the failure of these animals to thrive. In contrast, increased activity was demonstrated in the sevoflurane and isoflurane animals at 96 hours. The sevoflurane animals also showed increased rearing and decreased suckling scores at 48 and 96 hours. The clinical significance of this overall pattern is difficult to interpret. However, it most likely reflects the distinct recovery characteristics of the different volatile agents. Since sevoflurane is more rapidly cleared than other anesthetics, patients recover faster from sevoflurane anesthesia, and this may account for the increased agitation and rearing observed since, in humans, sevoflurane is associated with distinct emergence agitation, particularly in children [15]. While the agitation behavior we observed in sevoflurane- and isoflurane-treated animals was measured days after anesthetic recovery and could not be considered a true emergence agitation per se, it is likely that similar neurochemical signaling perturbances are involved in the manifestation of these analogous human and rodent behaviors. Decreased suckling, which was seen at both 48 and 96 h in the sevoflurane-exposed group and at 96 h in the isoflurane-exposed group, is a known behavioral indicator of alcohol-induced neurodevelopmental toxicity in young rats [46].

We studied the effect of these volatile anesthetics on neuroapoptosis on postnatal day 15, somewhat later than many previous reports. One previous report did examine synaptic alterations induced by a 2-hour anesthetic exposure to isoflurane, sevoflurane, and desflurane on postnatal day 16 [47]. It found no difference in neuroapoptosis but significantly increased dendritic spine density, suggesting that different volatile anesthetics can interfere with physiologic synaptogenesis to potentially impair the neuronal circuit assembly during postnatal development. Although we did not evaluate synaptogenesis here, the lack of neuroapoptosis in Briner's study might be attributable to a reduced insult arising from a shorter exposure time (2 h versus 4 h used in our study).

Numerous studies have shown that many anesthetics, and other drugs currently in clinical use, can produce widespread apoptosis in the developing rodent brain [8, 11, 20, 21, 31]. This has obviously caused significant concern amongst physicians as to whether they may be administering potential neurotoxins to fetuses and infants. The possibility that a routine anesthetic may be neurologically damaging to such patients is indeed alarming. This has to be balanced against the potential adverse consequences of withholding anesthesia, sedation, and/or surgery in the neonate where relevant pathology arises [48].

Anand and Soriano question whether these animal findings are attributable to the effects of anesthetics or whether other clinical factors (surgery, starvation, hypoxia, etc.) may be influential and whether any of these findings in rat and mice pups can be extrapolated to humans [49, 50]. They argue that systemic effects of anesthesia may be contributory, for example, anesthesia-induced hypotension could cause neurotoxicity. Because of the extremely small size of these rat pups, physiological monitoring and hemodynamic stabilization under anesthesia were not possible in our study. Of note, however, when hypoxic/ischemic neurodegeneration is induced in the neonatal rat brain, the acute cell death that ensues is not apoptotic but excitotoxic [51].

It is clear that a multidisciplinary effort, such as that epitomized by the SmartTots collaboration between the International Anaesthesia Research Society and the US FDA, is required to determine exactly how safe anesthetics are in human infants and children [52]. Moreover, high-quality randomized trials are required [53]. There is some ongoing clinical progress, such as the PANDA study, which is comparing neuropsychological outcomes between children under 3 years who received anesthesia for hernia repair and siblings who never received anesthesia. Only pilot data have so far been reported, and it failed to find any significant difference in verbal IQ, performance IQ, or full IQ in 28 sibling pairs aged 6–11 from this cohort, although a larger cohort may obviously yield different results [54].

With respect to the dosing regimen used in this study, we attempted to approximate the clinical setting throughout and importantly only subjected the animals to a single dose of drug. We exposed the animals to only 1.0 MAC volatile agent; however, it could be argued that the 4-hour exposure time, despite being similar or markedly reduced in comparison with other relevant studies, may have been excessive, as the PD15 rat brain has a greatly accelerated rate of cell turnover when compared to the human infant [8]. Indeed, rats at this age have neuroanatomical similarities to 2-month-old humans [55].

## 5. Conclusions

In this rat model of the neonatal developing brain, we have shown that volatile anesthetics are neurotoxic to the piriform cortex using behavioral and histochemical techniques, but that this effect is significantly less marked and may recover completely with sevoflurane. Further experimental and clinical studies are warranted to fully understand the mechanism of this effect in order to minimize it and to identify those drugs that have the least potential for clinical neurotoxicity.

## Figures and Tables

**Table 1:** Behavioral parameters of neonatal rats exposed to the anesthetic agents sevoflurane and isoflurane at 1 MAC for 4 h on postnatal day 15 and sacrificed 48 or 96 h later.

Behavioral parameter	Time (h)	Control	Sevoflurane	Isoflurane	Urethane	*P* value
Locomotion/activity score	48	9.9 ± 1.66 (*n*=10)	11.7 ± 2.70 (*n*=9)	9.38 ± 3.21 (*n*=8)	5.50 ± 1.32 (*n*=4)	0.03^*∗*^
Locomotion/activity score	96	11.2 ± 2.41 (*n*=12)	15.8 ± 1.56^†^ (*n*=9)	15.4 ± 2.03^†^ (*n*=88)	6.78 ± 2.17^*∗*^ (*n*=9)	0.02^*∗*^ 0.045^†^
Rearing score	48	2.60 ± 0.68	6.78 ± 1.04^†^	3.38 ± 0.78	1.25 ± 0.25^*∗*^	0.03^*∗*^ 0.01^†^
Rearing score	96	3.67 ± 0.68	7.56 ± 1.21^†^	4.25 ± 0.64	4.11 ± 1.31	0.01^†^
Suckling score	48	3.20 ± 0.53	1.67 ± 0.33^*∗*^	2.63 ± 0.37	3.00 ± 1.08	0.04^*∗*^
Suckling score	96	2.42 ± 0.36	0.89 ± 0.26^*∗*^	1.13 ± 0.23	4.56 ± 0.71	0.04^*∗*^
Grooming score	48	2.80 ± 0.53	2.22 ± 0.43	2.5 ± 0.57	1.50 ± 1.19^*∗*^	0.04^*∗*^

Animals were assessed behaviorally immediately prior to sacrifice as described in Methods. The *P* values for parameters that differed significantly are shown (^*∗*^significantly decreased from control; ^†^significantly increased above control). Numbers of animals per group are shown for locomotion data and apply to all behavioral parameters.
